# Comparison of genetic susceptibility to lung adenocarcinoma and squamous cell carcinoma in Japanese patients using a novel panel for cancer-related drug-metabolizing enzyme genes

**DOI:** 10.1038/s41598-022-22914-6

**Published:** 2022-10-26

**Authors:** Sumiko Ohnami, Akane Naruoka, Mitsuhiro Isaka, Maki Mizuguchi, Sou Nakatani, Fukumi Kamada, Yuji Shimoda, Ai Sakai, Keiichi Ohshima, Keiichi Hatakeyama, Kouji Maruyama, Yasuhisa Ohde, Hirotsugu Kenmotsu, Toshiaki Takahashi, Yasuto Akiyama, Takeshi Nagashima, Kenichi Urakami, Shumpei Ohnami, Ken Yamaguchi

**Affiliations:** 1 Cancer Diagnostics Research Division, Shizuoka Cancer Center Research Institute, 1007 Shimonagakubo, Nagaizumi-Cho, Shizuoka Japan; 2 Drug Discovery and Development Division, Shizuoka Cancer Center Research Institute, Nagaizumi, Shizuoka Japan; 3 Medical Genetics Division, Shizuoka Cancer Center Research Institute, Nagaizumi, Shizuoka Japan; 4 Cancer Multiomics Division, Shizuoka Cancer Center Research Institute, Nagaizumi, Shizuoka Japan; 5 Experimental Animal Facility, Shizuoka Cancer Center Research Institute, Nagaizumi, Shizuoka Japan; 6 Immunotherapy Division, Shizuoka Cancer Center Research Institute, Nagaizumi, Shizuoka Japan; 7 Division of Thoracic Surgery, Shizuoka Cancer Center Hospital, Nagaizumi, Shizuoka Japan; 8 Division of Thoracic Oncology, Shizuoka Cancer Center Hospital, Nagaizumi, Shizuoka Japan; 9 SRL, Inc, Tokyo, Japan; 10 Shizuoka Cancer Center, Nagaizumi, Shizuoka Japan

**Keywords:** Cancer, Oncology, Risk factors

## Abstract

The differences in genetic susceptibility to lung adenocarcinoma and squamous cell carcinoma remain unclear. We developed a customized, targeted gene sequencing panel for efficient and sensitive identification of germline variants, including whole-gene deletion types for cancer-related drug-metabolizing enzyme genes in lung adenocarcinoma and squamous cell carcinoma. The minor allele frequencies of the variants, confirmed as clinically significant in the Japanese population, did not differ significantly from those of normal participants listed in the public database. Genotype analysis comparing lung adenocarcinoma (n = 559) and squamous cell carcinoma (n = 151) indicated that the variants of *DPYD* (rs190771411, Fisher’s exact test, *P* = 0.045; rs200562975, *P* = 0.045) and *ALDH2* (rs568781254, *P* = 0.032) were associated with an increased risk of squamous cell carcinoma compared to adenocarcinoma. Conversely, whole-gene deletion of *CYP2A6* was associated with adenocarcinoma but not squamous cell carcinoma. Notably, whole-gene deletion of *CYP2A6* was confirmed in 22 patients with lung adenocarcinoma but not in any patients with squamous cell carcinoma. Most patients with whole-gene deletion of *CYP2A6* were female non-smokers. The discovery of a whole-gene deletion of *CYP2A6* in patients with lung adenocarcinoma may have an important role in clinical practice and advance our understanding of *CYP2A6* germline variants and their association with carcinogenesis or their susceptibility to lung adenocarcinoma.

## Introduction

Individual and racial differences exist in the occurrence of adverse effects of therapeutic drugs, including anticancer drugs. Therefore, detecting variants of genes encoding drug-metabolizing enzymes is vital for understanding the variations in drug response and individual risks of adverse effects^1–3^.

Additionally, various genetic damages induced by endogenous compounds and exogenous hazards, such as environmental chemicals, may contribute to the etiology of cancer^4^. Approximately 30% of drug-metabolizing enzyme substrates can be metabolically enhanced^5^. Some genetic variants of drug-metabolizing enzymes correlate with cancer risk. However, contradictory findings have also been reported. Phase I drug-metabolizing enzymes such as cytochrome P450 (CYPs), encoded by P450 genes, metabolize pro-carcinogens into genotoxic electrophilic intermediates. Phase II drug-metabolizing enzymes bind intermediates to water-soluble derivatives to complete the detoxification cycle. Therefore, the activity and expression of genes encoding phase I and phase II drug-metabolizing enzymes are important factors in defining the toxicity or carcinogenicity of environmental chemicals, including cancer susceptibility and smoking effects^4,6^.

Lung cancer is one of the cancers most strongly associated with exposure to environmental factors, such as smoking and inhalation of exhaust fumes. The overall landscape of genomic abnormalities in somatic cells of lung adenocarcinoma^7^ and squamous cell lung carcinoma, the most common subtypes of lung cancer, has been largely revealed^8,9^. The mutations in lung cancer cells of smokers mainly consist of cytosine to adenine (C > A) nucleotide transversions, which arise due to the mutagenic effect of tobacco. In contrast, non-smokers usually present a predominant transition from cytosine to thymine (C > T)^7^. Moreover, they have fewer somatic mutations and genomic breakpoints, and a smaller fraction of the genome with chromosomal instability than smokers^10^. Smoking is more strongly associated with squamous cell carcinoma than adenocarcinoma. However, in terms of genetic predisposition, the difference between lung adenocarcinoma and squamous cell carcinoma in germline variants of drug-metabolizing enzymes remains unclear.

Widespread use of next-generation sequencing has enabled comprehensive investigation of genetic variants, such as drug-metabolizing enzymes, using whole-genome sequencing (WGS) and whole-exome sequencing (WES). However, genes with high homologies, such as *CYP* genes, still have unanalyzable genetic variants^11,12^. Therefore, we constructed a unique genetic variant panel that mainly covers the exon regions of 20 genes, including both lifestyle- and cancer-related genes, focusing on drug-metabolizing enzyme-coding genes that influence the therapeutic and adverse effects of anticancer drugs. Here, we compared the differences in genetic susceptibility to lung adenocarcinoma and squamous cell carcinoma in the germline of Japanese patients using a novel panel (DME panel) and next-generation sequencing.

## Results

The total number of variants of the 20 target genes detected using the DME panel was 433 (Supplementary Fig. S1). The mean depth of coverage of the target regions was 455-fold that of the DME panel. All previously described to affect drug responses in Japanese populations were detectable among these genetic variants. The minor allele frequencies (MAFs) of the variants did not differ significantly from those of normal participants listed in the public database, suggesting that the DME panel is useful for comprehensively detecting germline mutations (Table 1).

**Table 1:** List of the genetic variants recognized as clinically significant genes in the Japanese population.

Gene symbol	NCBI SNP ID (rs number)	Reference (major)/variant (minor) allele	Amino acid residue change	Nucleotide exchange	MAF in Japanese^a^	MAF in this panel
*ABCG2*	rs2231142	C/A	Gln141Lys	421C > A	0.2967	0.3042
*ABCG2*	rs72552713	C/T	Gln126end	376C > T	0.0227	0.0212
*CYP1A2*	rs72547517	G/A	Arg456His	1367G > A	0.0062	0.0050
*CYP2A6*	rs8192720	C/T	L8L	22C > T	0.2490	0.2441
*CYP2B6*	rs3745274	G/T	Gly172His	523G > T	0.1685	0.1901
*CYP2B6*	rs8192709	C/T	Arg22Cys	64C > T	0.0572	0.0562
*CYP2C9*	rs1057910	A/C	Ile359Leu	1100A > C	0.0242	0.0243
*CYP2C19*	rs4986893	G/A	Trp212end	661G > A	0.1295	0.1266
*CYP2C19*	rs4244285	G/A	Pro227Pro	681G > A	0.2944	0.2943
*CYP2D6*	rs3892097	C/T	splicing	C > T	0.0007	0.0011
*CYP2E1*	rs2515641	T/C	Phe421Phe	1263T > C	0.8358	0.8273
*CYP3A4*	rs12721627	C/G	Thr185Ser	554C > G	0.0210	0.0218
*CYP3A5*	rs28365085	T/C	Ile488Thr	1463T > C	0.0124	0.0126
*CDA*	rs60369023	G/A	Ala70Thr	208G > A	0.0415	0.0388
*CDA*	rs2072671	A/C	Lys27Gln	79A > C	0.1968	0.1903
*COMT*	rs4680	G/A	Val158Met	721G > A	0.3125	0.3145
*DPYD*	rs188052243	A/G	Asn893Ser	2678A > G	0.0023	0.0028
*DPYD*	rs2297595	A/G	Met166Val	496A > G	0.0218	0.0152
*NAT2*	rs1801280	T/C	Ile114Thr	341T > C	0.0134	0.0150
*NAT2*	rs1799931	G/A	Gly286Glu	964G > A	0.0877	0.0977
*TPMT*	rs1142345	A/G	Tyr240Cys	896A > G	0.0096	0.0096
*UGT1A1*	rs4148323	G/A	Gly71Arg	226G > A	0.1740	0.1790
*ADH1B*	rs1229984	A/C	His48Pro	143A > C	0.2378	0.2254
*ALDH2*	rs671	G/A	Glu504Lys	1510G > A	0.2386	0.2637
*MTHFR*	rs1801131	A/C	Glu470Ala	1409A > C	0.1996	0.1874
*MTRR*	rs1801394	A/G	Ile22Met	66A > G	0.3019	0.3143

^a^MAF (minor allele frequency) is imformation from a Japanese database (HGVD or jMorp).

The characteristics of patients with adenocarcinoma and squamous cell carcinoma of the lungs are shown in Table 2. The number of patients with squamous cell carcinoma who smoked was significantly higher (*P* < 0.001) than that of patients with adenocarcinoma. The proportion of patients with squamous cell carcinoma (73.5%, 111/151) who consumed alcohol was also significantly higher (*P* < 0.001) than that of patients with adenocarcinoma (55.4%, 309/558).

**Table 2:** Characteristics of the patients with lung cancer.

Total number		Lung cancer		
		710		
		AD^a^	SCC^b^	*P* value^c^
		559	151	
**Gender**				
Male		294	131	
Female		265	20	< 0.001
**Age**				
≤ 50		17	1	
51–60		69	8	
61–70		206	62	
≥ 71		267	80	
**Smoking status**				
Nonsmokers		232	0	
Smokers		327	151	< 0.001
**Pack-years**^**d**^				
Light smokers (0 < to < 20)		77	6	
Heavy smokers (> 20)		248	145	< 0.001
Smokers but pack-years unknown		2	0	
**Drinking status**				
Nondrinkers		249	40	
Drinkers		309	111	< 0.001
Unknown		1	0	
**TNM stage (UICC TNM 7th)**				
pStage	IA	210	42	
	IB	159	42	
	IIA	55	24	
	IIB	43	23	
	IIIA	78	16	
	IIIB	1	2	
	IIIC	1	0	
	IV	12	2	
**Surgical procedure**				
Lobectomy		500	126	
Sublobar resection		59	25	
**Histologic patterns (subtypes) of adenocarcinoma**				
Acinar		230		
Mucinous		41		
Lepidic		116		
Papillary		82		
Solid		63		
Others^e^		19		
Unknown		8		
**Adjuvant therapy**				
Chemotherapy		112	22	
Radiotherapy		8	2	
Chemoradiotherapy		2	0	
**Family history of cancer**				
	Yes	390	96	
	No	108	35	
	Unknown	61	20	

^a^AD; Adenocarcinoma, ^b^SCC; Squamous cell carcinoma, ^c^*P* value by Fisher's exact test, ^d^Pack-years; defined as the number of packs of cigarettes amoked per day times of years of smoking, ^e^Others were as follows: minimally invasive (n = 15), moderately differentiated (n = 1), poorly differentiated (n = 2), and pulmonary (n = 1).

**Figure 1:**
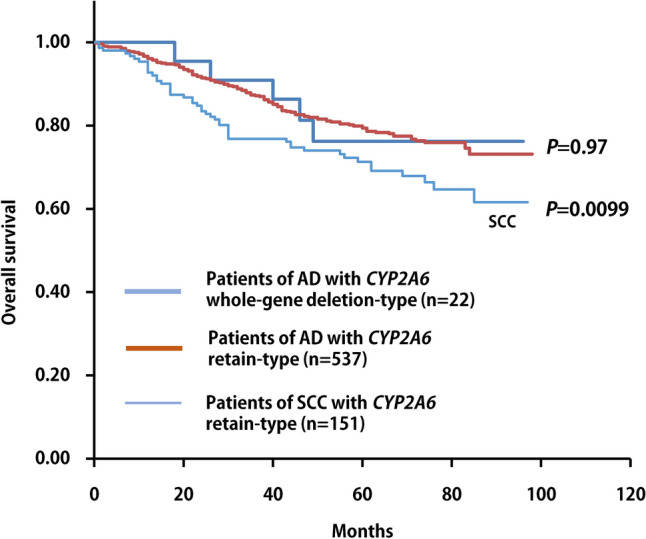
Kaplan–Meier survival curves for patients with or without whole-gene deletion-type of *CYP2A6* in lung adenocarcinoma and squamous cell carcinoma with *CYP2A6* retain-type.

The association analysis results of individual variants of squamous cell carcinoma and adenocarcinoma of the lungs are shown in Supplementary Table S1. Two variants of *DPYD* (rs190771411 and rs200562975) and a variant of *ALDH2* (rs568781254) were associated with an increased risk of squamous cell carcinoma compared to adenocarcinoma in the dominant model (*P* < 0.05) (Table 3). The characteristics of all 7 squamous cell carcinoma patients with significant variants in *DPYD* and *ALDH2* are shown in Table 4. No distinctive items were noted. Notably, a whole-gene deletion of *CYP2A6* was detected in 22 patients with adenocarcinoma but in no patient with squamous cell carcinoma (Table 5, Supplementary Fig. S2). In addition, 63.6% (14/22) of patients with a *CYP2A6* whole-gene deletion were non-smokers, and 72.7% (16/22) were women. To assess its clinical effect, we analyzed the effect of the *CYP2A6* whole-gene deletion in lung adenocarcinoma on overall survival (OS) using the Kaplan–Meier method. Patients with the *CYP2A6* whole-gene deletion-type showed no significant (*p* = 0.97) difference in terms of OS compared to those with the *CYP2A6* gene retain-type. Lung adenocarcinoma patients with the *CYP2A6* gene retain-type had significantly (*p* = 0.0099) better OS compared with squamous cell carcinoma patients with the *CYP2A6* gene retain-type (Fig. 1). The characteristics of all 22 adenocarcinoma patients with deletion-type of *CYP2A6* gene are shown in Table 6. These patients with *CYP2A6* whole-gene deletion-type on survivals showed no relationship between surgical procedure and TNM stage.

**Table 3:** The genetic variants of *DPYD* and *ALDH2* show significantly different frequencies between adenocarcinoma and squamous cell carcinoma in patients with lung cancer.

	Genotype	AD^a^	SCC^b^	*P* value^c^
		n = 559	n = 151	
*DPYD*	AA	552	148	
rs190771411	AG	0	2	
	GG	0	0	
	AG + GG	0	2	0.045
	Missing	7	1	
*DPYD*	TT	554	148	
rs200562975	TC	0	2	
	CC	0	0	
	TC + CC	0	2	0.045
	Missing	5	1	
*ALDH2*	AA	549	145	
rs568781254	AC	1	3	
	CC	0	0	
	AC + CC	1	3	0.032
	Missing	9	3	

^a^*AD* adenocarcinoma, ^b^*SCC* squamous cell carcinoma, ^c^*P* value by Fisher’s exact test.

**Table 4:** Characteristics of all patients (n = 7) of lung squamouse cell carcinoma with *DPYD and ALDH2 variants.*

Variants^a^	Case	Gender	Age	Smoking status^b^	Drinking status	Surgical procedure	pStage	Family history	Survival time (month)
*DPYD* variant 1	1	Female	59	Heavy	Yes	Lobectomy	IIIA	Yes	74 (death)
*DPYD* variant 1	2	Male	73	Heavy	No	Lobectomy	IA	Yes	74 (alive)
*DPYD* variant 2	3	Male	69	Heavy	Yes	Lobectomy	IB	Yes	57 (alive)
*DPYD* variant 2	4	Male	60	Heavy	No	Lobectomy	IIA	Yes	46 (alive)
*ALDH2* variant 1	5	Male	78	Heavy	No	Lobectomy	IIIA	unknown	12 (death)
*ALDH2* variant 1	6	Male	72	Heavy	Yes	Sublobar resection	IA	Yes	84 (alive)
*ALDH2* variant 1	7	Male	71	Heavy	Yes	Lobectomy	IA	Yes	66 (alive)

^a^Variants; *DPYD* variant 1, variant2, and *ALDH2* variant1 indicate rs190771411 (A > G), rs200562975 (T > C), and rs568781254 (A > C), respectively.

^b^Smoking status; Light smokers (0 < to < 20), Heavy smokers (> 20), as shown in Table 2.

**Table 5:** Genetic variants of *CYP2A6* show significantly different frequencies between adenocarcinoma and squamous cell carcinoma in patients with lung cancer.

	Genotype	AD^a^	SCC^b^	*P* value^c^
*CYP2A6*	Present	537	151	
	Whole-gene deletion	22^d^	0	0.007

^a^*AD* adenocarcinoma, ^b^*SCC* squamous cell carcinoma, ^c^*P* value by Fisher’s exact test. ^d^Smoking status of patients with *CYP2A6* whole-gene deletion; Never = 14, Light = 3, Heavy = 5. ^d^Sex of patients with *CYP2A6* whole-gene deletion; Female = 16, Male = 6.

**Table 6:** Characteristics of all patients (n = 22) of lung adenocarcinoma with *CYP2A6* whole-gene deletion.

Cases	Gender	Age	Smoking status^a^	Drinking status	Surgical procedure	pStage	Subtype	Family history	Survival time (month)
1	Female	79	Never	No	Lobectomy	IA	Acinar	Yes	96 (alive)
2	Female	57	Never	Yes	Lobectomy	IB	Acinar	Yes	95 (alive)
3	Female	80	Never	No	Lobectomy	IB	Mucinous	Yes	92 (alive)
4	Male	86	Heavy	Yes	Lobectomy	IB	Minimally	Yes	88 (alive)
5	Female	73	Never	No	Sublobar resection	IA	Papillary	Unknown	85 (alive)
6	Male	67	Light	No	Sublobar resection	IA	Lepidic	Yes	84 (alive)
7	Female	81	Heavy	No	Sublobar resection	IB	Acinar	No	49 (death)
8	Female	77	Never	No	Lobectomy	IA	Lepidic	Yes	78 (alive)
9	Male	73	Heavy	No	Lobectomy	IIIA	Acinar	No	77 (alive)
10	Female	68	Heavy	No	Lobectomy	IIIA	Acinar	Yes	76 (alive)
11	Male	68	Never	Yes	Lobectomy	IB	Acinar	Yes	75 (alive)
12	Female	68	Never	No	Lobectomy	IIIA	Acinar	Unknown	69 (alive)
13	Female	74	Never	No	Lobectomy	IIIA	Acinar	Yes	46 (death)
14	Female	66	Light	No	Lobectomy	IB	Acinar	Yes	40 (death)
15	Female	59	Never	Yes	Lobectomy	IA	Acinar	No	57 (alive)
16	Female	67	Never	No	Lobectomy	IIA	Papillary	Yes	55 (alive)
17	Male	84	Heavy	No	Lobectomy	IIA	Papillary	Yes	52 (alive)
18	Female	76	Never	No	Lobectomy	IIA	Acinar	Yes	18 (death)
19	Female	83	Never	Yes	Lobectomy	IB	Unknown	Yes	49 (alive)
20	Female	82	Never	No	Lobectomy	IB	Acinar	Yes	45 (alive)
21	Female	78	Never	No	Lobectomy	IIIA	Acinar	No	26 (death)
22	Male	69	Light	Yes	Lobectomy	IA	Acinar	Yes	41 (alive)

^a^Smoking status; Light smokers (0 < to < 20), Heavy smokers (> 20), as shown in Table 2.

## Discussion

This study presented an efficient and sensitive analysis of genetic variants, including whole-gene deletion types for drug-metabolizing enzymes and environmental- or lifestyle-related factors. Multiplex long-range PCR amplification with locus-specific primers and next-generation sequencing was also adopted for library products unique in the DME panel because of their high sequence identities to other CYPs. For example, the sequences of *CYP2A6* and *CYP2D6* are > 90% identical to those of *CYP2A7* and *CYP2D7*, respectively. Although there are reports that genetic variants of *CYP2A6*, including whole-gene deletions*,* are associated with lung cancer risk^13^, differences in the risk for adenocarcinoma and squamous cell carcinoma of the lungs remain poorly understood. Notably, the *CYP2A6* whole-gene deletion was confirmed in 22 patients with lung adenocarcinoma but in no patients having squamous cell carcinoma. In addition, patients with whole-gene deletions were primarily female non-smokers. Our results suggest that in lung adenocarcinoma, this finding may be associated with the mechanisms of carcinogens different than those activated by *CYP2A6*. Ariyoshi et al. demonstrated that the *CYP2A6* whole-gene deletion was not found in male smokers among Japanese patients with squamous cell carcinoma (0 of 105)^14^, which is consistent with our results.

*CYP2A6* is an enzyme responsible for metabolizing of nicotine- and tobacco-specific carcinogens. Genetic variants of *CYP2A6* are associated with changes in the activity of the *CYP2A6* enzyme, which influences smoking effects and the rate at which some tobacco-specific carcinogens are metabolized, which subsequently determines the incidence of lung cancer. In smokers with lower *CYP2A6* activity, tobacco-specific nitrosamines are activated at lower levels, decreasing their exposure to these activated lung carcinogens^15^. Considering that the whole-gene deletion of *CYP2A6* is found only in lung adenocarcinoma, the potential role of *CYP2A6* germline variants in lung carcinogenesis is intriguing. Its role may be explained by the following. Individuals with *CYP2A6* whole-gene deletions may be less susceptible to smoking effects. Therefore, some patients may have developed lung adenocarcinomas through a pathway unrelated to the function of *CYP2A6,* regardless of smoking. Conversely, squamous cell carcinoma that develops in squamous epithelial cells may be directly affected by smoking in a dose-dependent manner while maintaining the function of the *CYP2A6* variants.

Heterozygous or homozygous *CYP2A6* deletions may be associated with a decreased occurrence of gastric cancer in females and decreased total cancer, including lung, colon, and gastric cancers in female non-smokers^16^. Adenocarcinoma is the most common subtype of primary lung cancer in women and is considered to be due to the later adoption of smoking by women^17^. Additionally, estrogen and its receptors have been identified as factors that increase the risk of lung adenocarcinoma^18,19^. The biological significance of *CYP2A6* whole-gene deletions in lung adenocarcinoma may be the modulation of the cancer phenotype, which requires further investigation and may enhance our understanding of the oncogenic mechanism of lung adenocarcinoma. However, it remains unclear how *CYP2A6* whole-gene deletions are involved in the development of lung adenocarcinoma and their interaction with xenobiotic organisms. Therefore, verifying its function using cell lines with downregulated or without *CYP2A6* expression is necessary. This is currently being investigated in our laboratory. A limitation of the present study is that the absence of the *CYP2A6* whole-gene deletion in patients with squamous cell carcinoma is debatable because our results were derived from a small hospital-based sample. Therefore, it will be necessary to verify the results using a larger sample.

In the present study, the *ALDH2* (rs568781254) or *DPYD* variants (rs190771411 and rs200562975) were associated with an increased risk of squamous cell carcinoma patients compared to adenocarcinoma. However, due to the low frequency of the minor allele of the variants (MAF of 0.0029 for *ALDH2* and MAF of 0.0014 for *DPYD*), these were not large enough to detect an association with squamous cell carcinoma. Previous Japanese studies noted that genetic variants in *ALDH2* are involved in ethanol metabolism, specifically associated with the risk of esophageal cancers. The carcinogenic metabolite acetaldehyde, an ingredient in tobacco smoke and/or alcohol, is detoxified by *ALDH2*. Matsuo et al. reported that the *ALDH2* variant interacted with cigarette smoking in the risk of lung cancer in Japanese^20^. Fluoropyrimidines (5-FU and its prodrug capecitabine) are widely used treat several types of cancer. Several studies have shown a link between reduced *DPYD* enzyme activity and increasing the risk of severe toxicity. A recent study has reported that the functional alterations of enzyme activities caused by *DYPD* variants were characterized^21^. The rs200562975of *DPYD* identified in the present study reportedly reduced enzymatic activity to less than 70% of wild-type in vitro^21^. However, none of the previous studies examined whether the *DPYD* variants contribute to the risk of lung cancer. Additionally, there is a lack of studies assessing the functional effect of most variants for DPYD in vivo, and inferring possible functions based on the variants is difficult. Further studies are needed to confirm our findings and expose the underlying molecular mechanism.

## Materials and methods

### Participants

This study was conducted using blood samples from Project HOPE initiated at the Shizuoka Cancer Center (SCC; Shizuoka, Japan). The objective of this project was to improve cancer therapy^22^. Blood samples for germline analysis were obtained from 710 patients with lung cancer (559 adenocarcinomas and 151 squamous cell carcinomas) intraoperatively at SCC Hospital, Shizuoka, Japan, between January 2014 and January 2020. We performed deep sequencing of a custom DME panel using intraoperative blood samples.

The Institutional Review Board of SCC approved all experimental protocols (Authorization No.: 25-33). Written informed consent was obtained from all patients participating in this study. All experiments using clinical samples were performed in accordance with the approved Japanese ethical guidelines^23^.

### Construction of an in-house custom DME panel

We analyzed the genes encoding *CYP* isoforms (*CYP1A2*, *CYP2A6*, *CYP2B6*, *CYP2C9*, *CYP2C19*, *CYP2D6, CYP2E1*, *CYP3A4*, and *CYP3A5*), thiopurine methyltransferase (*TPMT*), dihydropyrimidine dehydrogenase (*DPYD*), N-acetyltransferase 2 (*NAT2*), UDP glucuronosyltransferase family member A1 (*UGT1A1*), catechol-O-methyltransferase (*COMT*), ATP binding cassette subfamily G member 2 (*ABCG2*), cytidine deaminase (*CDA*), alcohol dehydrogenase 1B (*ADH1B*), aldehyde dehydrogenase 2 (*ALDH2*), 5-methyltetrahydrofolate-homocysteine methyltransferase reductase (*MTRR*), and methylenetetrahydrofolate reductase (*MTHFR*) in this study because the variants of these genes have been reported to affect drug response in Japanese populations^11,24^.

The allele frequencies of each gene were compared with those obtained from the following public Japanese population databases: Human Genetic Variation Database (HGVD)^25^ (http://www.genome.med.kyoto-u.ac.jp) and Japanese Multi Omics Reference Panel (jMorp)^12^ (https://jmorp.megabank.tohoku.ac.jp/202109/).

Genomic DNA was isolated from the buffy coats of blood samples using a QIAmp DNA Blood Kit (Qiagen, Hilden, Germany). All genetic variants were analyzed using an Illumina sequencer with multiplex long-range PCR assay and Nextera DNA Flex Library Prep kit (Illumina, San Diego, CA, USA). Briefly, 50–100 ng of DNA was amplified using long-range multiplex PCR with locus-specific primers and a GXL DNA polymerase with each primer set (Supplementary Table S2). The amplicon library was prepared using the Nextera DNA Flex Library Prep kit (Illumina), and the library DNA was quantified on TapeStation using the D5000 kit (Agilent Technologies, Santa Clara, CA, USA). The libraries were subsequently used for sequencing (Supplementary Fig. S3). The sequencing data was analyzed using the pipeline described in our previous report^26^ and the clinical sequencing data analysis integrator (csDAI) (Mizuho-ir.co.jp/solution/research/life/infodata/csdai/index.html). The genetic variants were visualized using the Integrative Genomics Viewer^27^.

### Statistical analyses

Fisher’s exact test, crude odds ratio (OR), and 95% confidence interval (CI) were employed to evaluate statistical differences in genotype distributions and allele frequencies of each variant between adenocarcinoma and squamous cell carcinoma in patients with lung cancer. To compare large biased populations, we performed a Fisher’s exact test A patient’s survival was analyzed using the Kaplan–Meier method and log-rank test. Statistical significance was defined at *P* < 0.05.

## Supplementary Information

Supplementary Information.

## Data Availability

The genotype data referenced during the current study are available in a public repository that is accessible through the NCBI (https://www.ncbi.nlm.nih.gov/), HGVD (https://www.hgvd.genome.med.kyoto-u.ac.jp/), jMorp (https://jmorp.megabank.tohoku.ac.jp/202109/), and PharmGKB (https://www.pharmgkb.org/) websites. The information on the variants between individual samples is described in Supplementary Table S1. The sequence information of the primer sets used in this study is listed in Supplementary Table S2. Although the somatic data and sample information from patients used in this study were submitted to the National Bioscience Database Center (NBDC) as ‘Controlled-Access Data’ (the accession number, hum0127 https://humandbs.biosciencedbc.jp/en/), the germline data analyzed during the current study are not available publicly, but are available from the corresponding author on reasonable request. However, all data and materials generated and/or analyzed during the current study are included in the supplementary information files of this article.
