# Supplementary material for: Comparison of genetic susceptibility to lung adenocarcinoma and squamous cell carcinoma in Japanese patients using a novel panel for cancer-related drug-metabolizing enzyme genes
Source: Sci Rep. 2022 Oct 26;12:17928. doi: 10.1038/s41598-022-22914-6 (PMC9606290; doi:10.1038/s41598-022-22914-6)
Supplement: Supplementary file 1 — Supplementary Information. [file 41598_2022_22914_MOESM1_ESM.pdf]

Supplementary Information

Comparison of genetic susceptibility to lung adenocarcinoma and squamous cell carcinoma in Japanese patients using a novel panel for cancer-related drug-metabolizing enzyme genes

Sumiko Ohnami, Akane Naruoka, Mitsuhiro Isaka, Maki Mizuguchi, Sou Nakatani, Fukumi Kamada, Yuji Shimoda, Ai Sakai, Keiichi Ohshima, Keiichi Hatakeyama, Kouji Maruyama, Yasuhisa Ohde, Hirotsugu Kenmotsu, Toshiaki Takahashi, Yasuto Akiyama, Takeshi Nagashima, Kenichi Urakami, Shumpei Ohnami, Ken Yamaguchi

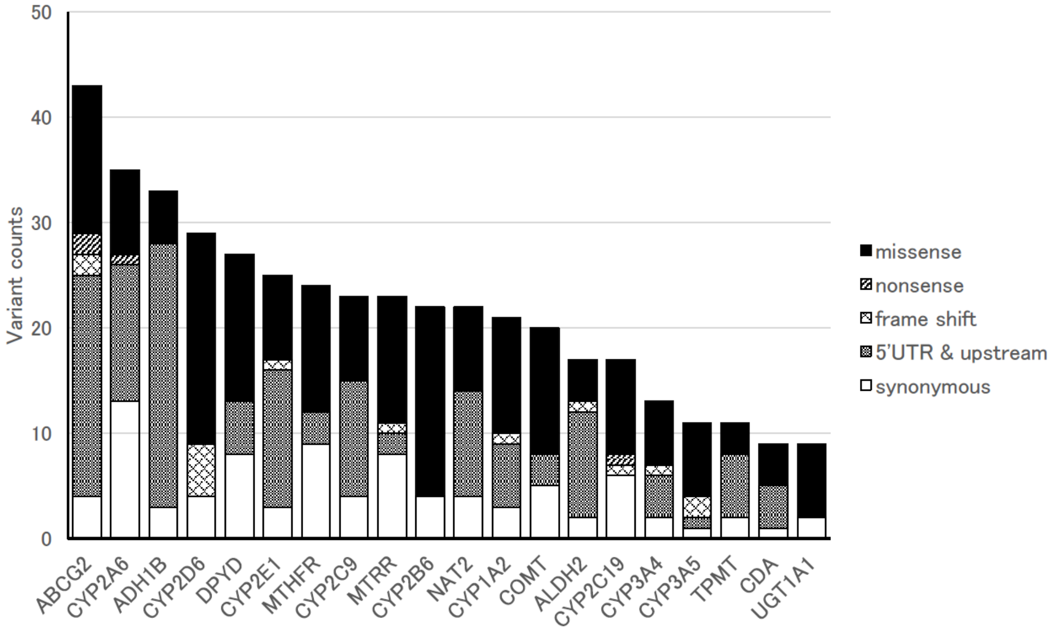

Supplementary Fig. S1

Distribution of genetic variants across the 20 drug-metabolizing enzymes in 710 cases.

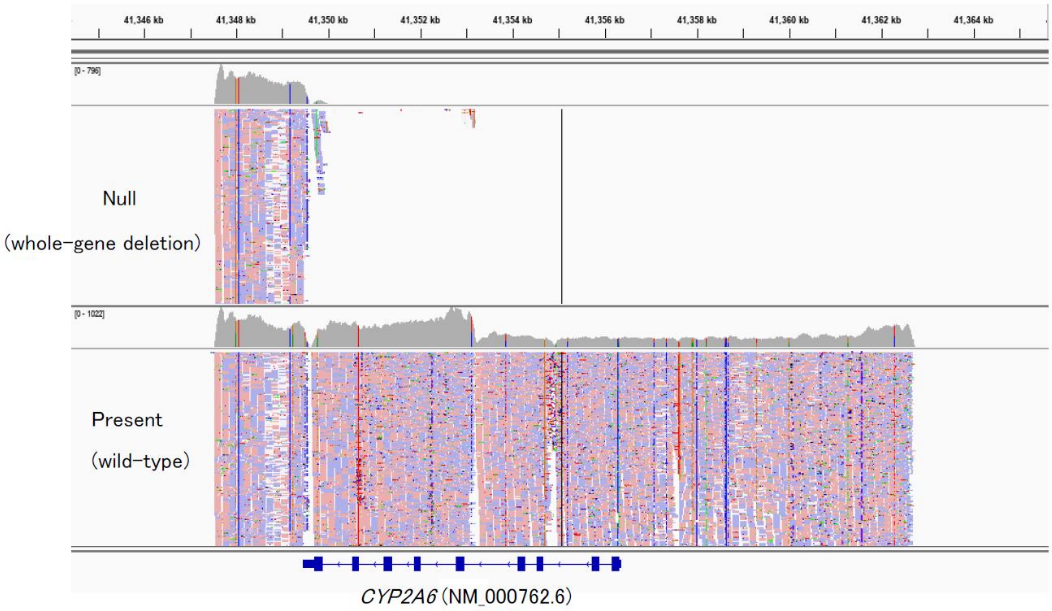

Supplementary Fig. S2

Visualization of the *CYP2A6* whole-gene deletion and wild-type calls using Integrative Genomics Viewer (IGV).

1.

Reagents		Final concentration
5x PrimeSTAR GXL Buffer		1x
dNTP	2.5 mM	0.2mM
GXL DNA Polymerase	1.25U/ μ l	0.625U
Template	50–100ng/ μ l	100–200ng
*Primer pool		0.2 μ M each
Nuclease-free water		
	Total volume	25 μ l

*Detailed statement of primer pools

Gene specific primers are constructed 113 primer sets

8 primer pools are designated as a, b, c, d, e, f, g, and h (lower Fig.A)

Size of expected PCRs are from 1,865bp to 9,545bp

↓

PCR

2.

Multiplex long-range PCR products

↓

Nextera DNA Flex Library Prep kit (Illumina)

↓

Library run on TapeStation (Agilent) using D5000 kit

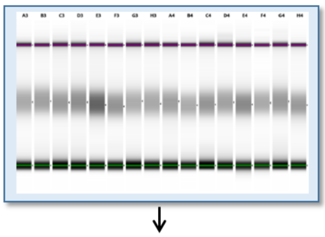

↓

NextSeq (Illumina) Sequencing

A.

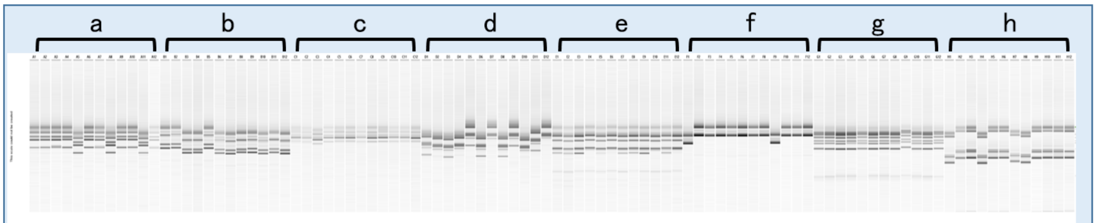

Confirmation of PCR products using QIAxcel Advanced System (Qiagen)

Supplementary Fig. S3

Multiplex long-range PCR method (1) and subsequent DNA library preparation and targeted sequencing using an Illumina sequencer (2). An indication of the electrophoresis pattern of long-range PCR.

Supplementary Table S1

Association of variants between adenocarcinoma and squamous cell carcinoma in lungs

	Gene symbol	rs No. or chr. position	nucleotide change	Adenocarcinoma (N=559)			Squamous cell carcinoma (N=151)			*P* value	OR (95%CI)
				AA	Aa	aa	AA	Aa	aa	AA vs Aa+aa	AA vs Aa+aa
1	*MTHFR*	rs2274976	C>T	476	75	4	133	17	1	0.508	0.82(0.47-1.41)
2	*MTHFR*	chr1:11852436	C>A	559	0	0	150	1	0	0.213	
3	*MTHFR*	rs750510348	G>A	550	4	0	148	1	0	1.000	
4	*MTHFR*	rs1801131	T>G	367	167	20	103	40	6	0.557	0.88(0.59-1.29)
5	*MTHFR*	chr1:11854881	G>A	554	0	0	148	1	0	0.212	
6	*MTHFR*	rs2066462	G>A	474	76	4	131	17	1	0.508	0.81(0.47-1.40)
7	*MTHFR*	rs755483936	C>T	557	1	0	151	0	0	1.000	
8	*MTHFR*	rs45498098	G>A	558	0	0	150	1	0	0.213	
9	*MTHFR*	rs781214043	C>T	557	1	0	151	0	0	1.000	
10	*MTHFR*	rs45459991	T>C	551	1	0	148	0	0	1.000	
11	*MTHFR*	rs1801133	G>A	200	250	101	55	68	25	0.848	0.96(0.66-1.40)
12	*MTHFR*	rs1018203291	C>T	549	2	0	148	0	0	1.000	
13	*MTHFR*	chr1:11861246	G>A	558	1	0	151	0	0	1.000	
14	*MTHFR*	chir1:11861247	C>A	558	1	0	151	0	0	1.000	
15	*MTHFR*	rs781158269	C>G	553	1	0	149	0	0	1.000	
16	*MTHFR*	rs201618781	C>T	553	1	0	149	0	0	1.000	
17	*MTHFR*	rs749729349	G>A	553	1	0	149	0	0	1.000	
18	*MTHFR*	rs2066470	G>A	461	90	3	130	18	1	0.258	0.72(0.42-1.23)
19	*MTHFR*	chr1:11863124	C>T	553	1	0	149	0	0	1.000	
20	*MTHFR*	rs768905024	G>A	552	2	0	149	0	0	1.000	
21	*MTHFR*	rs773463208	G>A	553	1	0	148	1	0	0.379	
22	*MTHFR*	rs764973403	A>G	554	0	0	148	1	0	0.212	
23	*MTHFR*	chr1:11865961	A>G	558	1	0	150	1	0	0.380	
24	*MTHFR*	chr1:11866090	G>A	559	0	0	150	1	0	0.213	
25	*CDA*	rs67820925	G>A	480	68	1	137	10	0	0.057	0.50(0.25-1.01)
26	*CDA*	rs602950	A>G	349	174	26	92	49	6	0.847	1.04(0.71-1.52)
27	*CDA*	rs3215400	C>-	178	262	109	47	65	35	1.000	1.02(0.69-1.50)
28	*CDA*	rs749081273	C>T	548	1	0	147	0	0	1.000	
29	*CDA*	rs777342792	G>A	548	1	0	147	0	0	1.000	
30	*CDA*	rs2072671	A>C	351	172	26	95	46	6	0.923	0.97(0.66-1.41)
31	*CDA*	rs201761717	C>T	557	1	0	150	0	0	1.000	
32	*CDA*	rs60369023	G>A	514	44	0	145	5	0	0.068	2.48(0.96-6.37)
33	*CDA*	rs76249360	T>C	551	7	0	148	2	0	1.000	
34	*DPYD*	rs1462320422	C>A	536	0	0	138	1	0	0.206	
35	*DPYD*	chr1:97564054	A>G	554	2	0	150	0	0	1.000	
36	*DPYD*	rs188052243	T>C	554	2	0	149	1	0	0.512	
37	*DPYD*	chr1:97658771	C>T	551	1	0	149	0	0	1.000	
38	*DPYD*	rs55725052	A>G	547	1	0	146	0	0	1.000	
39	*DPYD*	rs56005131	G>T	530	18	0	138	8	0	0.222	1.70(0.72-4.00)
40	*DPYD*	rs1801160	C>T	531	19	0	145	2	0	0.277	0.38(0.08-1.67)
41	*DPYD*	chr1:97847963	C>T	556	1	0	151	0	0	1.000	
42	*DPYD*	rs17376848	A>G	417	124	6	107	36	2	0.586	1.13(0.74-1.73)
43	*DPYD*	rs1801159	T>C	292	218	42	69	67	11	0.227	1.26(0.88-1.82)
44	*DPYD*	rs777368221	A>C	551	1	0	147	0	0	1.000	
45	*DPYD*	rs772264512	G>A	551	1	0	147	0	0	1.000	
46	*DPYD*	rs752985272	C>G	550	1	0	146	0	0	1.000	
47	*DPYD*	rs754745863	G>A	556	1	0	150	1	0	0.381	
48	*DPYD*	rs139863300	C>T	554	1	0	149	1	0	0.381	
49	*DPYD*	rs190048823	G>A	549	7	0	147	1	0	1.000	
50	*DPYD*	rs776973423	C>T	556	1	0	149	0	0	1.000	
51	*DPYD*	rs2297595	T>C	539	18	0	147	2	0	0.276	0.40(0.09-1.77)
52	*DPYD*	rs190771411	A>G	552	0	0	148	2	0	0.045	
53	*DPYD*	rs200562975	T>C	554	0	0	148	2	0	0.045	
54	*DPYD*	rs201841475	C>T	554	1	0	148	0	0	1.000	
55	*DPYD*	rs1801265	G>A	1	32	523	0	8	140	1.000	0.90(0.40-2.00)
56	*DPYD*	chr1:98386518	T>C	535	1	0	141	0	0	1.000	
57	*DPYD*	rs72981743	C>T	531	3	0	141	2	0	0.286	
58	*DPYD*	rs145248180	G>T	481	52	1	132	11	0	0.520	0.75(0.38-1.48)
59	*DPYD*	rs61787828	A>C	505	29	0	138	4	0	0.273	0.50(0.17-1.46)
60	*DPYD*	chr1:98386981	G>T	533	1	0	142	0	0	1.000	
61	*CYP2C19*	rs17885098	T>C	390	148	18	111	34	4	0.311	0.80(0.53-1.21)
62	*CYP2C19*	rs145328984	C>T	553	1	0	149	0	0	1.000	
63	*CYP2C19*	rs368767518	C>T	553	1	0	149	0	0	1.000	
64	*CYP2C19*	rs181297724	G>C	547	7	0	147	2	0	1.000	
65	*CYP2C19*	rs765456449	C>A	556	1	0	151	0	0	1.000	
66	*CYP2C19*	rs4986893	G>A	430	117	10	119	29	3	0.742	0.91(0.58-1.41)
67	*CYP2C19*	rs4244285	G>A	261	246	49	80	59	12	0.199	0.78(0.54-1.12)
68	*CYP2C19*	rs778258371	G>A	557	0	0	150	1	0	0.213	
69	*CYP2C19*	rs559628884	C>A	541	1	0	149	0	0	1.000	
70	*CYP2C19*	rs745503497	A/G	540	0	0	147	1	0	0.215	
71	*CYP2C19*	rs3758580	C>T	256	238	47	78	58	11	0.227	0.79(0.55-1.14)

72	*CYP2C19*	rs3758581	G>A	498	41	4	140	7	0	0.216	0.55(0.24-1.25)
73	*CYP2C19*	chr10:96602697	C>T	544	2	0	147	0	0	1.000	
74	*CYP2C19*	rs17886522	A>C	425	111	13	118	29	3	0.825	0.92(0.59-1.44)
75	*CYP2C19*	rs779501712	C>T	547	1	0	150	0	0	1.000	
76	*CYP2C19*	rs28399514	G>A	547	0	1	149	1	0	0.384	
77	*CYP2C19*	chr10:96612671	A>G	547	1	0	150	0	0	1.000	
78	*CYP2C9*	rs530860659	C>T	553	1	0	150	0	0	1.000	
79	*CYP2C9*	rs9332098	G>A	531	22	1	146	4	0	0.482	0.63(0.21-1.85)
80	*CYP2C9*	chr10:96697516	C>G	553	1	0	150	0	0	1.000	
81	*CYP2C9*	chr10:96697711	G>A	553	1	0	150	0	0	1.000	
82	*CYP2C9*	chr10:96697799	->T	548	6	0	149	1	0	1.000	
83	*CYP2C9*	rs397843768	T>-	553	1	0	150	0	0	1.000	
84	*CYP2C9*	chr10:96698030	T>C	553	1	0	150	0	0	1.000	
85	*CYP2C9*	rs1184084119	C>A	554	0	0	149	1	0	0.213	
86	*CYP2C9*	chr10:96698167	C>T	553	1	0	150	0	0	1.000	
87	*CYP2C9*	rs1309328671	A>G	552	2	0	150	0	0	1.000	
88	*CYP2C9*	chr10:96698425	AAG>-	553	1	0	150	0	0	1.000	
89	*CYP2C9*	rs114071557	A>G	553	1	0	150	0	0	1.000	
90	*CYP2C9*	rs17847036	G>A	544	13	0	150	0	0	0.082	
91	*CYP2C9*	rs201856860	T>C	555	2	0	149	1	0	0.512	
92	*CYP2C9*	rs12414460	G>A	555	2	0	150	0	0	1.000	
93	*CYP2C9*	rs1253395922	G>T	556	1	0	150	0	0	1.000	
94	*CYP2C9*	rs774550549	C>T	555	1	0	151	0	0	1.000	
95	*CYP2C9*	chr10:96707668	T>A	555	1	0	151	0	0	1.000	
96	*CYP2C9*	rs182132442	C>A	552	2	0	149	0	0	1.000	
97	*CYP2C9*	rs1057910	A>C	523	22	1	145	4	0	0.481	0.62(0.21-1.84)
98	*CYP2C9*	rs141283168	T>C	537	8	1	142	7	0	0.056	2.94(1.07-8.03)
99	*CYP2C9*	rs1057911	A>T	528	22	1	147	4	0	0.481	0.62(0.21-1.83)
100	*CYP2C9*	rs781583846	G>A	549	2	0	151	0	0	1.000	
101	*CYP2E1*	rs2031921	T>C	362	165	22	97	42	7	1.000	0.97(0.66-1.43)
102	*CYP2E1*	chr10:135339913	T>C	546	1	0	147	0	0	1.000	
103	*CYP2E1*	rs3813870	A>G	331	189	31	81	56	10	0.299	1.22(0.84-1.76)
104	*CYP2E1*	chr10:135340070	A>T	548	1	0	146	0	0	1.000	
105	*CYP2E1*	rs2031922	T>C	362	165	22	96	43	7	1.000	1.00(0.68-1.48)
106	*CYP2E1*	rs1219486901	G>A	545	1	0	146	0	0	1.000	
107	*CYP2E1*	rs2070672	A>G	332	189	31	81	56	9	0.344	1.21(0.83-1.75)
108	*CYP2E1*	rs2070673	A>T	98	261	193	30	72	44	0.471	
109	*CYP2E1*	chr10:135340594	A>G	545	2	0	146	0	0	1.000	
110	*CYP2E1*	chr10:135340995	G>A	546	1	0	145	0	0	1.000	
111	*CYP2E1*	rs72559710	G>A	555	2	0	151	0	0	1.000	
112	*CYP2E1*	chr10:135342066	A>G	556	1	0	151	0	0	1.000	
113	*CYP2E1*	chr10:135345205	C>T	546	1	0	147	0	0	1.000	
114	*CYP2E1*	rs141280653	C>T	546	1	0	147	0	0	1.000	
115	*CYP2E1*	rs778316659	G>A	546	1	0	147	0	0	1.000	
116	*CYP2E1*	rs2070674	C>T	365	158	22	96	40	11	0.695	1.07(0.73-1.58)
117	*CYP2E1*	rs28371742	C>G	533	12	0	145	2	0	0.745	0.61(0.13-2.76)
118	*CYP2E1*	rs566294180	A>G	544	2	0	147	0	0	1.000	
119	*CYP2E1*	rs41299426	A>G	545	1	0	147	0	0	1.000	
120	*CYP2E1*	rs1390074994	G>C	546	0	0	146	1	0	0.212	
121	*CYP2E1*	chr10:135347317	G>C	545	1	0	147	0	0	1.000	
122	*CYP2E1*	rs28371746	C>A	517	27	1	138	9	0	0.679	1.20(0.55-2.61)
123	*CYP2E1*	rs199856651	A>G	552	4	0	148	2	0	0.613	0.67(0.04-11.29)
124	*CYP2E1*	rs2515641	T>C	17	144	395	5	43	102	0.481	1.15(0.78-1.70)
125	*CYP2E1*	chr10:135352305	A>-	555	1	0	150	0	0	1.000	
126	*ALDH2*	chr12:112203692	T>A	551	0	0	147	1	0	0.212	
127	*ALDH2*	chr12:112203720	T>C	550	1	0	148	0	0	1.000	
128	*ALDH2*	rs568781254	A>C	549	1	0	145	3	0	0.032	11.3(1.17-110.0)
129	*ALDH2*	rs551263044	C>T	549	1	0	148	0	0	1.000	
130	*ALDH2*	rs1480473723	C>T	549	1	0	148	0	0	1.000	
131	*ALDH2*	rs1414849157	C>T	548	2	0	148	0	0	1.000	
132	*ALDH2*	rs886205	A>G	32	166	352	12	49	87	0.251	1.24(0.86-1.80)
133	*ALDH2*	chr12:112204571	G>A	549	1	0	148	0	0	1.000	
134	*ALDH2*	rs991114674	G>A	549	1	0	148	0	0	1.000	
135	*ALDH2*	chr12:112204719	G>A	549	1	0	148	0	0	1.000	
136	*ALDH2*	rs141574314	A>G	522	1	0	142	0	0	1.000	
137	*ALDH2*	rs13306164	C>T	509	12	0	139	3	0	1.000	
138	*ALDH2*	chr12:112229894	CAGGTTGCTGCTGGG>-	557	1	0	151	0	0	1.000	
139	*ALDH2*	chr12:112230450	G>A	556	0	0	150	1	0	0.214	
140	*ALDH2*	rs1331843613	G>A	534	1	0	146	0	0	1.000	
141	*ALDH2*	rs671	G>A	280	228	46	73	65	11	0.782	1.06(0.74-1.52)
142	*CYP1A2*	chr15:75040196	T>G	548	1	0	0	147	0	1.000	
143	*CYP1A2*	rs2069523	G>T	548	1	0	147	0	0	1.000	
144	*CYP1A2*	rs2069524	A>G	512	37	0	141	6	0	0.334	0.58(0.24-1.42)
145	*CYP1A2*	rs2069525	T>C	496	51	1	138	9	0	0.251	0.62(0.29-1.29)
146	*CYP1A2*	chr15:75040864	G>C	555	4	0	150	1	0	1.000	
147	*CYP1A2*	rs143211242	A>C	557	2	0	151	0	0	1.000	
148	*CYP1A2*	rs941101723	G>A	558	1	0	151	0	0	1.000	
149	*CYP1A2*	rs3743482	G>A	559	0	0	150	1	0	0.213	
150	*CYP1A2*	rs752037611	G>A	559	0	0	150	1	0	0.213	

151	*CYP1A2*	rs138652540	C>T	557	2	0	151	0	0	1.000	
152	*CYP1A2*	rs773366123	T>A	559	0	0	150	1	0	0.213	
153	*CYP1A2*	rs56037745	G>A	556	2	0	151	0	0	1.000	
154	*CYP1A2*	rs35796837	G>A	555	3	0	150	1	0	1.000	
155	*CYP1A2*	rs55918015	G>A	554	4	0	151	0	0	0.583	
156	*CYP1A2*	rs56107638	G>A	557	1	0	150	1	0	0.381	
157	*CYP1A2*	rs45486893	C>T	539	16	0	148	3	0	0.778	
158	*CYP1A2*	rs72547517	G>A	547	7	0	150	1	0	1.000	
159	*CYP1A2*	rs199528490	G>C	552	2	0	151	0	0	1.000	
160	*CYP1A2*	chr15:75047379	C>T	553	1	0	151	0	0	1.000	
161	*CYP1A2*	rs111900570	C>T	554	1	0	150	1	0	0.382	
162	*CYP1A2*	rs2470890	C>T	348	180	27	101	44	5	0.339	0.81(0.55-1.19)
163	*CYP2A6*	CYP2A6(*4/*4)	CYP2A6>deletion	537	0	22	151	0	0	0.007	
164	*CYP2A6*	rs539466327	G>A	534	1	0	151	0	0	1.000	
165	*CYP2A6*	rs143731390	T>A	533	2	0	150	1	0	0.526	
166	*CYP2A6*	rs2002977	G>A	522	11	3	147	2	2	1.000	
167	*CYP2A6*	rs143336165	G>A	534	2	0	150	1	0	0.526	
168	*CYP2A6*	rs28399462	G>A	535	0	1	150	0	1	0.392	
169	*CYP2A6*	rs56314118	A>G	535	1	0	151	0	0	1.000	
170	*CYP2A6*	rs59389036	G>A	535	1	0	151	0	0	1.000	
171	*CYP2A6*	rs561177628	G>A	535	1	0	151	0	0	1.000	
172	*CYP2A6*	rs28399455	G>A	535	1	0	151	0	0	1.000	
173	*CYP2A6*	rs2644907	G>C	533	3	0	151	0	0	1.000	
174	*CYP2A6*	rs140471703	C>T	534	2	0	151	0	0	1.000	
175	*CYP2A6*	rs1809811	G>A	524	11	2	148	2	1	1.000	
176	*CYP2A6*	rs28399447	A>G	520	15	2	150	1	0	0.144	0.20(0.02-1.54)
177	*CYP2A6*	rs199916117	T>C	501	4	0	136	1	0	1.000	
178	*CYP2A6*	rs72549434	T>C	522	4	2	144	2	0	0.686	
179	*CYP2A6*	rs1355151029	C>T	527	1	0	146	0	0	1.000	
180	*CYP2A6*	rs199545200	G>A	521	6	1	146	0	0	0.356	
181	*CYP2A6*	rs747840206	C>T	530	1	0	146	0	0	1.000	
182	*CYP2A6*	rs8192721	C>T	515	11	2	143	2	1	1.000	
183	*CYP2A6*	rs1137115	T>C	38	104	385	7	31	108	0.916	0.95(0.62-1.44)
184	*CYP2A6*	rs8192720	G>A	356	118	53	96	37	13	0.691	1.08(0.73-1.59)
185	*CYP2A6*	rs28399434	C>T	516	8	2	146	0	0	0.129	
186	*CYP2A6*	rs28399433	A>C	323	135	70	97	32	17	0.289	0.79(0.54-1.17)
187	*CYP2A6*	rs535847711	C>T	532	0	0	145	1	0	0.215	
188	*CYP2A6*	chr19:41356623	T>G	531	1	0	146	0	0	1.000	
189	*CYP2A6*	chr19:41356790	T>G	527	0	0	145	1	0	0.217	
190	*CYP2A6*	rs371984288	T>C	525	3	0	146	0	0	1.000	
191	*CYP2A6*	rs1357380496	C>A	527	0	1	146	0	0	1.000	
192	*CYP2A6*	chr19:41356848	G>A	532	0	0	145	1	0	0.215	
193	*CYP2A6*	rs539519931	G>A	526	2	0	146	0	0	1.000	
194	*CYP2A6*	chr19:41356946	C>T	527	1	0	146	0	0	1.000	
195	*CYP2A6*	rs61663607	T>C	348	121	58	93	38	15	0.623	1.10(0.75-1.62)
196	*CYP2A6*	rs1452707033	C>G	524	1	0	146	0	0	1.000	
197	*CYP2A6*	rs1439083763	G>T	521	2	0	146	0	0	1.000	
198	*CYP2A6*	rs4803381	T>C	131	166	226	38	43	65	0.830	0.94(0.62-1.44)
199	*CYP2B6*	rs8192709	C>T	476	76	3	136	12	1	0.099	0.57(0.31-1.06)
200	*CYP2B6*	rs367708702	G>A	537	0	0	145	1	0	0.214	
201	*CYP2B6*	chr19:41509928	T>C	535	1	0	146	0	0	1.000	
202	*CYP2B6*	rs2279341	G>C	457	74	3	133	11	1	0.053	0.53(0.28-1.01)
203	*CYP2B6*	rs757472041	C>T	536	1	0	146	0	0	1.000	
204	*CYP2B6*	rs375508968	C>T	536	1	0	146	0	0	1.000	
205	*CYP2B6*	rs3826711	C>G	530	8	0	142	4	0	0.296	1.86(0.55-6.28)
206	*CYP2B6*	rs3745274	G>T	378	142	20	90	45	11	0.058	1.45(0.99-2.12)
207	*CYP2B6*	rs565530668	C>T	558	0	0	150	1	0	0.213	
208	*CYP2B6*	rs139029625	G>A	557	1	0	151	0	0	1.000	
209	*CYP2B6*	rs142806708	G>C	555	2	0	151	0	0	1.000	
210	*CYP2B6*	rs201500445	T>C	557	0	0	150	1	0	0.213	
211	*CYP2B6*	rs200238771	T>A	556	1	0	151	0	0	1.000	
212	*CYP2B6*	rs35661880	C>T	554	3	0	148	3	0	0.115	
213	*CYP2B6*	rs147991149	C>T	557	0	0	150	1	0	0.213	
214	*CYP2B6*	rs3211369	A>G	552	5	0	151	0	0	0.590	
215	*CYP2B6*	rs373442191	G>A	556	1	0	151	0	0	1.000	
216	*CYP2B6*	rs117872433	G>A	557	0	0	150	1	0	0.213	
217	*CYP2B6*	rs200993638	C>T	552	5	0	151	0	0	0.590	
218	*CYP2B6*	rs3211370	A>T	552	5	0	151	0	0	0.590	
219	*CYP2B6*	rs564083989	G>A	556	1	0	151	0	0	1.000	
220	*CYP2B6*	rs3211371	C>T	544	13	0	146	5	0	0.558	1.43(0.50-4.08)
221	*UGT1A1*	rs4148323	G>A	343	148	20	92	38	6	1.000	0.97(0.65-1.46)
222	*UGT1A1*	rs200412341	C>T	518	0	0	134	1	0	0.207	
223	*UGT1A1*	rs1029804751	G>A	516	1	0	135	0	0	1.000	
224	*UGT1A1*	rs35350960	C>A	516	2	0	134	1	0	0.501	
225	*UGT1A1*	rs34946978	C>T	549	4	0	146	1	0	1.000	
226	*UGT1A1*	rs201427749	C>T	550	1	0	148	0	0	1.000	
227	*UGT1A1*	rs115944950	G>C	551	0	0	147	1	0	0.212	
228	*UGT1A1*	rs34993780	T>G	548	3	0	146	2	0	0.287	
229	*UGT1A1*	rs867885761	G>C	550	1	0	147	1	0	0.379	

230	*COMT*	chr22:19948728	C>T	554	1	0	149	0	0	1.000	
231	*COMT*	rs558998170	A>G	552	3	0	148	1	0	1.000	
232	*COMT*	rs1171801279	G>T	554	1	0	149	0	0	1.000	
233	*COMT*	rs74745580	C>T	557	2	0	151	0	0	1.000	
234	*COMT*	rs1261617944	T>C	558	1	0	151	0	0	1.000	
235	*COMT*	rs4633	C>T	282	224	53	67	73	11	0.200	1.27(0.88-1.83)
236	*COMT*	rs6267	G>T	463	93	3	131	20	0	0.267	0.73(0.43-1.23)
237	*COMT*	rs373611092	A>G	557	2	0	151	0	0	1.000	
238	*COMT*	rs76452330	G>A	532	27	0	146	5	0	0.513	1.48(0.56-3.91)
239	*COMT*	rs199710929	C>T	558	1	0	151	0	0	1.000	
240	*COMT*	rs4818	C>G	280	236	43	67	73	11	0.233	1.25(0.87-1.80)
241	*COMT*	rs201835143	C>T	557	2	0	151	0	0	1.000	
242	*COMT*	rs4680	G>A	277	225	57	64	72	15	0.120	1.33(0.92-1.91)
243	*COMT*	rs1460479393	G>A	558	1	0	151	0	0	1.000	
244	*COMT*	rs769224	G>A	515	43	1	141	9	1	0.730	0.83(0.40-1.69)
245	*COMT*	rs4646315	G>C	425	120	14	117	31	3	0.747	0.92(0.60-1.41)
246	*COMT*	rs201656482	A>C	549	10	0	151	0	0	0.131	
247	*COMT*	chr22:19956087	T>C	558	1	0	151	0	0	1.000	
248	*COMT*	rs749437638	C>T	559	0	0	150	1	0	0.213	
249	*COMT*	rs201407028	G>A	558	1	0	151	0	0	1.000	
250	*CYP2D6*	rs1440526469	C>T	555	1	0	149	0	0	1.000	
251	*CYP2D6*	rs1135840	G>C	168	253	135	52	62	34	0.273	0.79(0.54-1.17)
252	*CYP2D6*	rs765776661	-/CAGTGGGCA	554	1	1	149	0	0	1.000	
253	*CYP2D6*	rs1135835	T>C	553	3	0	149	0	0	1.000	
254	*CYP2D6*	rs1372322657	G>A	555	1	0	149	0	0	1.000	
255	*CYP2D6*	rs28371733	C>T	555	1	0	149	0	0	1.000	
256	*CYP2D6*	rs747089665	G>A	555	1	0	149	0	0	1.000	
257	*CYP2D6*	rs769157652	C>T	555	0	1	149	0	0	1.000	
258	*CYP2D6*	rs141739595	G>A	556	0	0	148	1	0	0.211	
259	*CYP2D6*	rs76088846	C>T	556	0	0	148	1	0	0.211	
260	*CYP2D6*	chr22:42523850	C>T	555	1	0	149	0	0	1.000	
261	*CYP2D6*	chr22:42523883	A>G	555	1	0	149	0	0	1.000	
262	*CYP2D6*	rs16947	A>G	23	141	392	3	35	111	0.360	1.22(0.81-1.84)
263	*CYP2D6*	rs1135829	T>C	554	2	0	148	1	0	0.510	
264	*CYP2D6*	rs72549352	->G	545	7	2	147	1	0	0.697	0.41(0.05-3.27)
265	*CYP2D6*	rs749182114	G>T, G>C	552	2	0	148	0	0	1.000	
266	*CYP2D6*	rs28371718	G>T	528	0	0	139	1	0	0.210	
267	*CYP2D6*	chr22:42524247	G>A	554	0	0	148	1	0	0.212	
268	*CYP2D6*	rs72549353	AGTT>-	553	1	0	149	0	0	1.000	
269	*CYP2D6*	rs3892097	C>T	554	2	0	149	0	0	1.000	
270	*CYP2D6*	rs5030865	C>T	551	5	0	148	1	0	1.000	
271	*CYP2D6*	rs1058164	G>C	164	239	124	49	57	34	0.415	0.83(0.56-1.24)
272	*CYP2D6*	rs1135822	A>T	519	8	1	139	1	0	0.697	0.41(0.05-3.30)
273	*CYP2D6*	chr22:42525739	C>T	521	1	0	139	0	0	1.000	
274	*CYP2D6*	rs1081003	G>A	220	215	86	54	55	30	0.499	1.15(0.78-1.68)
275	*CYP2D6*	chr22:42525787	G>T	552	0	0	147	1	0	0.211	
276	*CYP2D6*	rs28371705	G>C	548	4	0	148	0	0	0.584	
277	*CYP2D6*	rs1065852	G>A	235	222	92	57	58	32	0.398	1.18(0.81-1.71)
278	*CYP2D6*	rs267608313	G>A	550	1	0	148	0	0	1.000	
279	*ABCG2*	rs763740151	G>A	557	1	0	150	0	0	1.000	
280	*ABCG2*	rs748531218	T>C	555	3	0	150	0	0	1.000	
281	*ABCG2*	rs759701118	G>A	550	1	0	147	0	0	1.000	
282	*ABCG2*	rs868217328	G>-	550	1	0	147	0	0	1.000	
283	*ABCG2*	rs192169063	A>G	544	7	0	146	1	0	1.000	
284	*ABCG2*	rs1354553769	C>T	550	1	0	147	0	0	1.000	
285	*ABCG2*	rs35622453	C>T	522	12	0	140	4	0	0.757	1.24(0.39-3.91)
286	*ABCG2*	rs140207606	G>A	512	0	0	137	1	0	0.212	
287	*ABCG2*	chr4:89042907	T>C	521	1	0	141	0	0	1.000	
288	*ABCG2*	chr4:89052224	C>T	555	0	1	150	0	0	1.000	
289	*ABCG2*	chr4:89052257	T>C	555	1	0	150	0	0	1.000	
290	*ABCG2*	rs769012528	C>T	555	1	0	150	0	0	1.000	
291	*ABCG2*	rs201006821	C>G	555	1	0	150	0	0	1.000	
292	*ABCG2*	rs2231142	G>T	268	239	49	61	71	18	0.117	1.35(0.94-1.95)
293	*ABCG2*	rs149106245	T>A	555	1	0	149	1	0	0.380	
294	*ABCG2*	rs72552713	G>A	528	26	1	142	8	0	0.832	1.10(0.48-2.47)
295	*ABCG2*	rs2231139	G>A	552	2	0	150	0	0	1.000	
296	*ABCG2*	rs199473672	G>A	551	4	0	149	1	0	1.000	
297	*ABCG2*	rs146682354	T>C	556	1	0	151	0	0	1.000	
298	*ABCG2*	rs1319203095	G>A	556	1	0	151	0	0	1.000	
299	*ABCG2*	rs2231137	C>T	356	181	20	90	57	4	0.343	1.20(0.83-1.73)
300	*ABCG2*	rs4148151	C>A	556	1	0	151	0	0	1.000	
301	*ABCG2*	chr4:89079596	G>C	544	1	0	149	0	0	1.000	
302	*ABCG2*	rs2231136	G>A	537	10	0	143	5	0	0.334	0.53(0.17-1.58)
303	*ABCG2*	chr4:89079887	C>T	545	1	0	148	0	0	1.000	
304	*ABCG2*	chr4:89079919	G>C	544	2	0	147	1	0	0.514	
305	*ABCG2*	rs1400423296	C>T	554	3	0	151	0	0	1.000	
306	*ABCG2*	rs72554040	G>A	274	231	52	68	73	10	0.409	1.18(0.82-1.69)
307	*ABCG2*	rs182367277		550	7	0	147	4	0	0.260	0.46(0.13-1.61)
308	*ABCG2*	rs186907101		557	0	0	150	1	0	0.213	

309	*ABCG2*	rs1334731718	C>T	554	3	0	151	0	0	1.000	
310	*ABCG2*	rs62309960	G>C	540	15	2	149	2	0	0.393	0.42(0.09-1.86)
311	*ABCG2*	rs546576886	G>A	544	13	0	149	2	0	0.749	
312	*ABCG2*	rs766442580	C>T	556	1	0	151	0	0	1.000	
313	*ABCG2*	rs36111742	A>G	549	8	0	149	2	0	1.000	
314	*ABCG2*	chr4:89152786	CTGAGGGT>-	556	1	0	151	0	0	1.000	
315	*ABCG2*	rs542537786	A>-	555	2	0	150	1	0	0.514	
316	*ABCG2*	rs62309962	A>C	540	15	2	149	2	0	0.393	0.42(0.09-1.86)
317	*ABCG2*	rs7681726	G>A	0	8	548	0	2	149	1.000	
318	*ABCG2*	rs562002915	T>C	545	11	1	148	3	0	1.000	
319	*ABCG2*	chr4:89153301	->A	556	1	0	151	0	0	1.000	
320	*ABCG2*	chr4:89153381	A>T	556	1	0	151	0	0	1.000	
321	*ABCG2*	chr4:89153436	T>C	556	1	0	151	0	0	1.000	
322	*ADH1B*	rs150939574	T>C	553	1	0	151	0	0	1.000	
323	*ADH1B*	rs74451421	C>T	552	2	0	150	1	0	0.515	
324	*ADH1B*	rs1789882	A>G	4	68	482	0	11	140	0.063	0.52(0.27-1.01)
325	*ADH1B*	chr4:100235107	G>C	555	1	0	151	0	0	1.000	
326	*ADH1B*	rs1181667845	C>A	554	1	0	151	0	0	1.000	
327	*ADH1B*	rs750389182	A>C	554	1	0	151	0	0	1.000	
328	*ADH1B*	rs772635726	T>G	556	0	0	149	1	0	0.212	
329	*ADH1B*	rs1229984	T>C	341	176	39	99	41	10	0.342	0.81(0.55-1.19)
330	*ADH1B*	chr4:100240130	T>C	556	0	0	149	1	0	0.212	
331	*ADH1B*	rs1440331692	C>T	555	1	0	150	0	0	1.000	
332	*ADH1B*	rs28913910	TGG>-	550	5	0	148	2	0	0.645	
333	*ADH1B*	rs993180442	C>T	553	2	0	150	0	0	1.000	
334	*ADH1B*	chr4:100240742	G>A	554	1	0	149	1	0	0.381	
335	*ADH1B*	rs1258284878	G>A	553	3	0	150	0	0	1.000	
336	*ADH1B*	chr4:100240879	A>G	554	1	0	150	0	0	1.000	
337	*ADH1B*	chr4:100240976	A>G	555	1	0	150	0	0	1.000	
338	*ADH1B*	chr4:100241124	G>A	555	1	0	150	0	0	1.000	
339	*ADH1B*	chr4:100241340	T>C	556	0	0	149	1	0	0.212	
340	*ADH1B*	rs1353621	T>C	533	23	1	143	7	0	0.824	1.08(0.45-2.57)
341	*ADH1B*	chr4:100241826	G>A	555	1	0	150	0	0	1.000	
342	*ADH1B*	chr4:100241832	C>A	555	1	0	150	0	0	1.000	
343	*ADH1B*	chr4:100242490	T>A	555	1	0	150	0	0	1.000	
344	*ADH1B*	chr4:100242813	->T	553	2	0	149	1	0	0.513	
345	*ADH1B*	rs927053190	C>T	554	1	0	150	0	0	1.000	
346	*ADH1B*	rs1302702778	A>G	553	2	0	149	1	0	0.513	
347	*ADH1B*	rs1159918	A>C	12	126	416	3	26	121	1.000	0.92(0.25-3.30)
348	*ADH1B*	chr4:100243086	C>T	554	1	0	150	0	0	1.000	
349	*ADH1B*	rs573647108	A>T	554	1	0	150	0	0	1.000	
350	*ADH1B*	rs2070898	G>A	16	144	396	4	31	115	1.000	0.92(0.30-2.80)
351	*ADH1B*	rs2070897	G>A	12	127	417	3	26	121	1.000	0.92(0.25-3.32)
352	*ADH1B*	rs6810842	T>G	12	127	418	3	25	122	1.000	0.92(0.25-3.32)
353	*ADH1B*	rs28913903	G>C	247	247	63	74	61	15	0.310	0.81(0.57-1.17)
354	*ADH1B*	rs1034245804	G>T	555	1	0	150	0	0	1.000	
355	*MTRR*	rs1182239347	G>A	552	0	0	147	1	0	0.211	
356	*MTRR*	rs72716536	T>C	382	157	13	104	39	5	0.841	0.95(0.63-1.41)
357	*MTRR*	rs778827432	G>A	550	1	0	147	0	0	1.000	
358	*MTRR*	rs1355632408	T>G	550	1	0	147	0	0	1.000	
359	*MTRR*	rs148267849	G>T	548	1	0	147	0	0	1.000	
360	*MTRR*	rs114748706	T>C	535	14	0	143	4	0	1.000	
361	*MTRR*	rs1801394	A>G	237	254	54	73	58	16	0.192	0.78(0.54-1.12)
362	*MTRR*	rs149300444	G>A	546	1	0	146	0	0	1.000	
363	*MTRR*	chr5:7875409	G>A	556	1	0	149	0	0	1.000	
364	*MTRR*	rs577851734	T>C	556	1	0	148	1	0	0.378	
365	*MTRR*	rs1532268	C>T	429	119	6	112	34	2	0.660	1.10(0.72-1.68)
366	*MTRR*	rs161870	T>C	409	140	5	105	40	3	0.531	
367	*MTRR*	rs752182014	A>G	552	2	0	148	0	0	1.000	
368	*MTRR*	rs201557658	C>T	552	2	0	148	0	0	1.000	
369	*MTRR*	rs2303080	T>A	428	121	5	117	29	2	0.739	0.90(0.57-1.40)
370	*MTRR*	rs162036	A>G	398	136	5	105	39	3	0.599	1.12(0.75-1.69)
371	*MTRR*	rs2287779	G>A	375	159	9	109	29	6	0.125	0.71(0.46-1.09)
372	*MTRR*	rs2287780	C>T	372	162	8	109	30	6	0.153	0.72(0.47-1.09)
373	*MTRR*	rs16879334	C>G	368	156	8	106	30	6	0.216	0.76(0.50-1.16)
374	*MTRR*	rs139206262	G>A	543	1	0	147	0	0	1.000	
375	*MTRR*	rs10380	C>T	423	120	2	110	34	3	0.508	1.16(0.76-1.78)
376	*MTRR*	rs12347	G>A	422	118	2	110	34	3	0.439	1.18(0.77-1.80)
377	*MTRR*	rs1802059	G>A	417	119	6	109	36	2	0.512	1.16(0.76-1.76)
378	*TPMT*	rs1142345	T>C	510	12	0	134	4	0	0.755	1.26(0.40-3.99)
379	*TPMT*	rs2842949	C>A	46	179	284	13	49	71	0.739	1.09(0.57-2.08)
380	*TPMT*	rs75543815	T>A	516	4	0	138	1	0	1.000	
381	*TPMT*	rs2842934	G>A	48	193	290	15	48	84	0.633	1.14(0.62-2.10)
382	*TPMT*	rs750424422	C>T	554	1	0	151	0	0	1.000	
383	*TPMT*	rs376768623	A>T	555	1	0	151	0	0	1.000	
384	*TPMT*	rs747281890	C>T	555	1	0	151	0	0	1.000	
385	*TPMT*	rs141028204	G>T	531	2	0	141	0	0	1.000	
386	*TPMT*	rs1256925667	G>C	531	1	0	141	1	0	0.377	
387	*TPMT*	rs1002196683	G>T	527	1	0	141	0	0	1.000	

388	*TPMT*	rs1279888446	A>G	523	5	0	142	0	0	0.590	
389	*CYP3A5*	rs749945666	G>T	547	2	0	148	0	0	1.000	
390	*CYP3A5*	rs28365085	A>G	536	14	0	143	5	0	0.572	1.33(0.47-3.77)
391	*CYP3A5*	rs776843927	G>A	549	1	0	148	0	0	1.000	
392	*CYP3A5*	rs756271054	T>C	550	0	0	147	1	0	0.212	
393	*CYP3A5*	rs761366357	C>T	559	0	0	150	1	0	0.213	
394	*CYP3A5*	chr7:99261713	A>G	558	1	0	151	0	0	1.000	
395	*CYP3A5*	rs56411402	T>C	558	1	0	151	0	0	1.000	
396	*CYP3A5*	rs1445725863	G>T	558	1	0	151	0	0	1.000	
397	*CYP3A5*	rs55965422	A>G	557	2	0	151	0	0	1.000	
398	*CYP3A5*	rs759812876	C>A	558	1	0	151	0	0	1.000	
399	*CYP3A5*	chr7:99277540	T>G	557	1	0	150	0	0	1.000	
400	*CYP3A4*	rs147125009	G>A	543	0	0	146	1	0	0.213	
401	*CYP3A4*	rs142425279	T>C	542	1	0	146	0	0	1.000	
402	*CYP3A4*	rs1309748501	G>A	544	2	0	148	0	0	1.000	
403	*CYP3A4*	rs28371759	A>G	549	10	0	149	2	0	1.000	
404	*CYP3A4*	rs34784390	->T	553	1	0	149	0	0	1.000	
405	*CYP3A4*	rs55901263	G>C	557	1	0	150	1	0	0.381	
406	*CYP3A4*	rs12721627	G>C	536	22	0	149	2	0	0.134	3.05(0.71-13.1)
407	*CYP3A4*	rs55951658	T>C	555	2	0	151	0	0	1.000	
408	*CYP3A4*	chr7:99370267	G>A	557	1	0	151	0	0	1.000	
409	*CYP3A4*	rs55679469	A>G	553	1	0	150	0	0	1.000	
410	*CYP3A4*	rs36231115	C>T	551	3	0	149	1	0	1.000	
411	*CYP3A4*	rs1296063536	G>A	553	1	0	150	0	0	1.000	
412	*CYP3A4*	rs1298207919	T>C	552	2	0	149	1	0	0.513	
413	*NAT2*	rs934550523	G>T	525	1	0	140	0	0	1.000	
414	*NAT2*	rs4646267	A>G	114	262	149	31	78	30	0.908	0.96(0.61-1.51)
415	*NAT2*	rs55736132	A>G	488	35	1	125	15	0	0.152	1.62(0.86-3.06)
416	*NAT2*	rs4345600	A>G	332	178	15	91	45	4	0.767	0.92(0.62-1.36)
417	*NAT2*	rs4345601	A>T	332	178	15	91	45	4	0.767	0.92(0.62-1.36)
418	*NAT2*	rs35436619	->AATT	304	196	26	83	47	10	0.773	0.94(0.64-1.37)
419	*NAT2*	rs4271002	G>C	333	177	15	91	46	3	0.767	0.93(0.63-1.37)
420	*NAT2*	chr8:18248305	A>G	524	1	0	140	0	0	1.000	
421	*NAT2*	rs1426644302	AGAG>-	526	1	0	140	0	0	1.000	
422	*NAT2*	rs4646246	A>G	117	279	154	33	80	34	0.736	0.93(0.60-1.44)
423	*NAT2*	rs1805158	C>T	553	1	0	149	0	0	1.000	
425	*NAT2*	rs1041983	C>T	278	225	51	73	64	12	0.854	1.04(0.73-1.50)
426	*NAT2*	rs1801280	T>C	530	24	0	143	6	0	1.000	
427	*NAT2*	chr8:18257976	G>A	553	1	0	149	0	0	1.000	
428	*NAT2*	rs770612696	G>A	553	1	0	149	0	0	1.000	
429	*NAT2*	rs1799929	C>T	529	25	0	142	7	0	1.000	
430	*NAT2*	rs139512288	T>C	553	1	0	149	0	0	1.000	
431	*NAT2*	rs1799930	G>A	362	167	25	91	51	7	0.337	1.20(0.82-1.74)
432	*NAT2*	rs1309998351	G>A	553	1	0	149	0	0	1.000	
433	*NAT2*	rs1208	G>A	0	41	513	1	13	135	0.212	
434	*NAT2*	rs1799931	G>A	448	103	3	127	21	1	0.234	0.73(0.44-1.20)

Supplementary Table S2

The sequeces of the primer sets used in this study

Gene	Primer name	Forward: Primer sequence	Primer name	Reverse: Primer sequence	Product size (bp)
MTHFR	MTHFR_1_F	5'-TAAACGCGCTCTTGCCAGGGAA-3'	MTHFR_1_R	5'-CCTGTCAAACCAGTTGCTGC-3'	7784
MTHFR	MTHFR_2_F	5'-CACTTCCCCTCTCCTCCTTG-3'	MTHFR_2_R	5'-AGTTCGCTGAGTTCTTCCCA-3'	4546
MTHFR	MTHFR_3_F	5'-CCTGAGGCTGAGTTCTTCCA-3'	MTHFR_3_R	5'-AACTACTGTGGCCTGGAGAC-3'	6351
MTHFR	MTHFR_4_F	5'-GTCGAGGTCAAGAGGTGGAA-3'	MTHFR_4_R	5'-ATCGTGGGAGTTGGCTTACA-3'	4197
MTHFR	MTHFR_5_F	5'-CAGAACAAAAGCTGCCCCAT-3'	MTHFR_5_R	5'-GACCAGGCCACTCACTACTT-3'	3247
CDA	CDA_1_F	5'-CATCCTGAAAGCTGCGTACC-3'	CDA_1_R	5'-CACCATGCCCGACTATTGTG-3'	2088
CDA	CDA_2_F	5'-CACGGAACAAAATACTGCATAATCACAATG-3'	CDA_2_R	5'-GTTTGTGCTCACCTTGGTCACACACTGAGG-3'	6260
CDA	CDA_3_F	5'-ACCTGTGGTGCAGGCTTGGAATTTTCCATC-3'	CDA_3_R	5'-GAAGATGGAAGTGTCTGCATCCCCAGATGC-3'	5076
CDA	CDA_4_F	5'-ATGCAGACACTTCCATCTTCC-3'	CDA_4_R	5'-GTCTAGACAAACCGTGCTTG-3'	2822
DPYD	DPYD_1_F	5'-TTCCTTGCCTATCTCCCAGC-3'	DPYD_1_R	5'-TCTCAGAAGACCGGTGCTTT-3'	3014
DPYD	DPYD_2_F	5'-TCATCCTAAGTATTGAGAGAGTAGTAGCTG-3'	DPYD_2_R	5'-CAGAAGACCTGAAATATACAAAAGTGCCAA-3'	5012
DPYD	DPYD_3_F	5'-GTGCTTCTACATATGGTTCTTGAAGCATAA-3'	DPYD_3_R	5'-AGTGATTTGTAAACTCGGATACTGACAAGA-3'	5970
DPYD	DPYD_4_F	5'-GCCTAGTGAAAACTCTAAGTTGTTTTCTTG-3'	DPYD_4_R	5'-GTGTGTGTGTGTAGGGCTTTATTAAGATAT-3'	7131
DPYD	DPYD_5_F	5'-GATCCTCTGGATTTTCCTATGAATTTTAGG-3'	DPYD_5_R	5'-GGGAACAGGTGACTTTTTGTTACACTGGTA-3'	8013
DPYD	DPYD_6_F	5'-TGGTGATGCCATCCTCTGAG-3'	DPYD_6_R	5'-TGGGCAGGCAGTTTCCTAAA-3'	7211
DPYD	DPYD_7_F	5'-TTTGCTTCAGGAGATGCTGGGAAATGGATT-3'	DPYD_7_R	5'-CTGGGTTCTGGATATAATCAGGTTATGTTA-3'	4910
DPYD	DPYD_8_F	5'-CAGAGACTTTCATTTCTATCCTCTCTCAAT-3'	DPYD_8_R	5'-GTGCCTTCTCCCACAAATACCCACCTC-3'	5838
DPYD	DPYD_9_F	5'-ATCCGGTCAGCCACATTCTG-3'	DPYD_9_R	5'-CCGGCCTCCTTGAAAGACAA-3'	7492
DPYD	DPYD_10_F	5'-CATAGGGATGTGGTGATCAGGGAGCAGGTG-3'	DPYD_10_R	5'-GTTGAGGCACTTATCATTTTATGATAGGGA-3'	7271
DPYD	DPYD_11_F	5'-ACATAACAGAAAATCCCCCCCGTTTTATAA-3'	DPYD_11_R	5'-TAGAGAGTTACACAGCAGGAGGTAAGATTA-3'	8053
DPYD	DPYD_12_F	5'-TGGCCTTTTCTCCTTCGATTGA-3'	DPYD_12_R	5'-CAGATATTCATTTAGCCTCCTTTGGC-3'	7701
DPYD	DPYD_13_F	5'-GAATAAAGGCCCGTTTTTGCTATAAAGACA-3'	DPYD_13_R	5'-CCATTGGTAGGAGTTATTTTCCAGGAAAAT-3'	5125
DPYD	DPYD_14_F	5'-GCACTTGTTTTCCCTTCTTCCC-3'	DPYD_14_R	5'-ACCCCTTGCTCCTAAACCTC-3'	4559
DPYD	DPYD_15_F	5'-TTCTAGCTTCTCTGACTTGATGTTTTCCTT-3'	DPYD_15_R	5'-GCTGCCTAGATTCTCATTCATCAAAGTAAA-3'	7203
DPYD	DPYD_16_F	5'-TGCCATGCTCTGAGACAACA-3'	DPYD_16_R	5'-GTTGACTGCCTGTCCTGTTTT-3'	7226
DPYD	DPYD_17_F	5'-TGCCCATGGTCACATCTGAC-3'	DPYD_17_R	5'-ACCCCATGTAGATGAGGGCT-3'	7026
DPYD	DPYD_18_F	5'-AGGCCAGGTTTTCTGCTGAA-3'	DPYD_18_R	5'-TTTCTAACCATGGGGCCTGG-3'	6841
DPYD	DPYD_19_F	5'-CAATGCACAAAGAAAATCCTGTAGGAATCT-3'	DPYD_19_R	5'-AGGCTTATGTAACATGTAACACCAAGGACA-3'	5031
DPYD	DPYD_20_F	5'-ATTCCTTCCTGCTCGAGGGA-3'	DPYD_20_R	5'-CCCACCTACAAACAGACTGCA-3'	7248
CYP2C19	CYP2C19_1_F	5'-CCCGTCCCTTTCAGGTACAG-3'	CYP2C19_1_R	5'-TCACCGAATTCCCAACAGCA-3'	7529
CYP2C19	CYP2C19_2_F	5'-CAAGTGAAAGGGATCCAGTGGTACTTACTG-3'	CYP2C19_2_R	5'-TGGGGTTGTTTGTAGGAGATCCATCAGAGT-3'	5286
CYP2C19	CYP2C19_3_F	5'-CTGCTCAGGGAATTAAGAGATTACAAAAAC-3'	CYP2C19_3_R	5'-CTCTTTTCCATCACACACCTCTACACCTCC-3'	6275
CYP2C19	CYP2C19_4_F	5'-GCATCCACTACACCATACAGTATTTTTTCT-3'	CYP2C19_4_R	5'-GCAAATGTGCAAGTCTGAAAACACACTATT-3'	7547
CYP2C19	CYP2C19_5_F	5'-GGGTTATCTGATCTAAAATACCTAGGTCTA-3'	CYP2C19_5_R	5'-AAATGGCCAGAGTGTCTATTCACAGGGCAT-3'	8574
CYP2C19	CYP2C19_6_F	5'-GACACTCTGGCCATTTCCCA-3'	CYP2C19_6_R	5'-TTCCTCCCAATACCTGATCCTCT-3'	6500
CYP2C9	CYP2C9_1_F	5'-TGACCTTGATCAGCAATGGTTTTTAA-3'	CYP2C9_1_R	5'-TCCATGCAGCACCACTATGG-3'	7157
CYP2C9	CYP2C9_2_F	5'-CATCTTTTATTGCATCCACAACTGTGGTTC-3'	CYP2C9_2_R	5'-TTCCCATTTGAATACCCCCTTGACTGATTG-3'	7224
CYP2C9	CYP2C9_3_F	5'-CCTGACTTCCTGAAACATATAGATGTGAAT-3'	CYP2C9_3_R	5'-CAGGTTACATATGCTGTGAGTCCTTTATAT-3'	8143
CYP2C9	CYP2C9_4_F	5'-CAGTATCCTTACTCTCTCCTTTATCCCCTG-3'	CYP2C9_4_R	5'-GTACCCCCAAACATATTGATATATACCTAC-3'	5248
CYP2C9	CYP2C9_5_F	5'-CTCTAGATACGTGAACTTGTTTCTGAATTC-3'	CYP2C9_5_R	5'-AGTTGAAAGCCAGTCTTTGGAATCTTGTGC-3'	6243
CYP2C9	CYP2C9_6_F	5'-CAGCACTGTGTGGCCAATTC-3'	CYP2C9_6_R	5'-TCTCCTCCTCTGTCCAGGTG-3'	7659
CYP2E1	CYP2E1_1_F	5'-TGGAAGCTGCCTTTGTAGGG-3'	CYP2E1_1_R	5'-GGGGCCAGCAGAAGGTTATT-3'	7146
CYP2E1	CYP2E1_2_F	5'-CCGCCTACGACTGTTGTGAA-3'	CYP2E1_2_R	5'-TGGTTCCCCTAGTGCAGAGT-3'	7636
CYP2E1	CYP2E1_3_F	5'-GTGCCCCATCCCTGGCCTTTGCTTTTTCAA-3'	CYP2E1_3_R	5'-GTAGTGAAAGTTCCTAACAAAAGAGGAAGT-3'	5272
ALDH2	ALDH2_1_F	5'-CCTCATCTCTCTGCCTCTGG-3'	ALDH2_1_R	5'-GGGGCTTACTCCTGGAAGTT-3'	5095
ALDH2	ALDH2_2_F	5'-GGAACAGCTTGCTGGGAGAT-3'	ALDH2_2_R	5'-AGGATTTTCCCATGAGCTGG-3'	6027
ALDH2	ALDH2_3_F	5'-AGCTTTCTTCCAGGGTCACA-3'	ALDH2_3_R	5'-GACACGAAGGTTCACAGAGC-3'	4467
ALDH2	ALDH2_4_F	5'-TCCTGTCTTTCTGTCCCCAC-3'	ALDH2_4_R	5'-CGGAGGCTCTTACCACACAGT-3'	8037
ALDH2	ALDH2_5_F	5'-ACCACTCATGCACACACAGT-3'	ALDH2_5_R	5'-CAGCAAATGACCGCATAGGC-3'	7774
ALDH2	ALDH2_6_F	5'-ATGAGCCTCTGTATCCCCAC-3'	ALDH2_6_R	5'-TTTACCCACAGCAGAGGAGG-3'	3100
CYP1A2	CYP1A2_1_F	5'-GTTTCAAGCAAAGGGATCAGCTTTGGAGAA-3'	CYP1A2_1_R	5'-TGCTCACATGCTCCTCCAGGTAGCAGGAGG-3'	7566
CYP1A2	CYP1A2_2_F	5'-TCATCTAATCTCCAGTCCGTGCTTACATGT-3'	CYP1A2_2_R	5'-AGTTGATTCCTGTGCACTAAAGTTTGTGAA-3'	5299
CYP1A2	CYP1A2_3_F	5'-GTTACAGGTGATGGTTCCCC-3'	CYP1A2_3_R	5'-AGGAGAGGAGTGAAGGAGGT-3'	2995
CYP2A6	CYP2A6_1_F	5'-GTGCTCCAATCCAGCCTCGTTTAAATACCT-3'	CYP2A6_1_R	5'-AATATTGCTTCTTCTAATCTCACTGTGTCG-3'	5682
CYP2A6	CYP2A6_2_F	5'-AAAGTGTAATTCTTACCTAAGGAAAGGACC-3'	CYP2A6_2_R	5'-TATCCAGGTTTCAGGTATTTAAACGAGGCT-3'	9545
CYP2B6	CYP2B6_1_F	5'-AACACCATAGGTACAACTTCAGGGAAAATG-3'	CYP2B6_1_R	5'-TTACCAGGCCAACTACAAATGAATGTCTCT-3'	8514
CYP2B6	CYP2B6_2_F	5'-GCAGGGGTGGCTACAAGAAT-3'	CYP2B6_2_R	5'-CTTGCCCCGAATTCCTTCCT-3'	7694
CYP2B6	CYP2B6_3_F	5'-GTTTCATTCCAGCTGGGGTGTAGTGTTGCC-3'	CYP2B6_3_R	5'-TCTCTTAAAACAAATTAGCAGGCCAGTCGC-3'	7133
CYP2B6	CYP2B6_4_F	5'-GAAATAAGACAAAGGGCTCATCTGGGCAGG-3'	CYP2B6_4_R	5'-AAGCTTGTCTGTGGAATCCCACCCAAAGTA-3'	6178
UGT1A1	UGT1A1_1_F	5'-TTTCTACGCGTCCGACAACA-3'	UGT1A1_1_R	5'-AATAGCAGCAAGACGGGCAT-3'	7894
UGT1A1	UGT1A1_2_F	5'-CCCTGCCAGGGTAAGATAGCAGTGGTTCAC-3'	UGT1A1_2_R	5'-GCTGTGTTACTATTATCACCTGAAAGGTTA-3'	8097
UGT1A1	UGT1A1_3_F	5'-CCTAAGAACAACTTGGTGAAAGTCC-3'	UGT1A1_3_R	5'-GGGATGGTGGGAGCCATGTTTT-3'	5574
COMT	COMT_1_F	5'-CCAACTCTGCCCATTCACAC-3'	COMT_1_R	5'-CTACACGTCAGCCCTTACCA-3'	3343
COMT	COMT_2_F	5'-AGCCAGTTGTGGTAGCAGAC-3'	COMT_2_R	5'-GCCTCACCTTGGCCTATCAG-3'	6726
COMT	COMT_3_F	5'-AGTCCTCTGCCTCCTCCTT-3'	COMT_3_R	5'-GCTCGCAGTAGGTGTCAATG-3'	7912
COMT	COMT_4_F	5'-ACACACCTGCTCTGTCTACC-3'	COMT_4_R	5'-GTGTCTTTCCTGCCCAAGTG-3'	3656
COMT	COMT_5_F	5'-CCTTGTCATCCCAGAACCCT-3'	COMT_5_R	5'-GCACTGCATCCTCACTCATG-3'	5488
COMT	COMT_6_F	5'-GGGCAGAAAGTGGAAACCTG-3'	COMT_6_R	5'-ATCTGGTTGTTCGTCCACCT-3'	2809
CYP2D6	CYP2D6_1_F	5'-TATAGCTCCCTGACGCCATG-3'	CYP2D6_1_R	5'-CATTTCCCAGCTGGAATCCG-3'	3425
CYP2D6	CYP2D6_2_F	5'-TGGCCATGAAGGCATTAGCCCCA-3'	CYP2D6_2_R	5'-CGCCATCTCCAAAACAAAAGAAAAGCC-3'	6124
ABCG2	ABCG2_1_F	5'-CTGGGATTCAATGGCTATATTGCTAAATTC-3'	ABCG2_1_R	5'-AGAAACCCCAAAGCACAGAGATAGCCATCA-3'	5619
ABCG2	ABCG2_2_F	5'-GCACCAGGGCATCATTTTCT-3'	ABCG2_2_R	5'-TAACGGCCAGGTCTGAAAAC-3'	2105
ABCG2	ABCG2_3_F	5'-AGGAAGCACCATGTGGACAG-3'	ABCG2_3_R	5'-CAGGTTGCCTGTTATGCGTA-3'	4481
ABCG2	ABCG2_4_F	5'-CAGGGGCTAGGGATGGGATA-3'	ABCG2_4_R	5'-GGTGCATACAGATCTGGCCA-3'	7417
ABCG2	ABCG2_5_F	5'-TGGATGCTTCCCTAACACCT-3'	ABCG2_5_R	5'-CGGCTCTACCCAGTTCGAAT-3'	8938
ABCG2	ABCG2_6_F	5'-CAATAGACAGGCTATTTCTCTGTAGTTTTC-3'	ABCG2_6_R	5'-CTCATCCTACCCTCAATAAAAGAATGACAT-3'	7178
ABCG2	ABCG2_7_F	5'-CTTCCACATGACTTACATTCTTTGTTCAAC-3'	ABCG2_7_R	5'-GCAATCATAGAAAAGGCACGTGTTACTAAA-3'	8216

ABCG2	ABCG2_8_F	5'-CACTTGAATAAGTTGAGAAAAAAACCCCGT-3'	ABCG2_8_R	5'-GCTGCTCTTCTTGAAGGTAGTTGAAAAAAA-3'	5217
ADH1B	ADH1B_1_F	5'-CACCCAGCAACAGGTAGTGT-3'	ADH1B_1_R	5'-GCAGCCTTGACCTAGGACAG-3'	6519
ADH1B	ADH1B_2_F	5'-TATGCCCAAGGTAGAGGAGC-3'	ADH1B_2_R	5'-CTGTGGCAGGCATTGAAAGA-3'	5317
ADH1B	ADH1B_3_F	5'-AGTCGAGAATCCACAGCCAA-3'	ADH1B_3_R	5'-CCACAAAAGAGAATGGGCCC-3'	7201
MTRR	MTRR_1_F	5'-TGAGAGAACACTGGTCCACC-3'	MTRR_1_R	5'-GAGAACACTGCCCTTACCCT-3'	7557
MTRR	MTRR_2_F	5'-AGACTTTACCCCAGAGCCAC-3'	MTRR_2_R	5'-GCTGCCATGAACTGATCCTG-3'	5608
MTRR	MTRR_3_F	5'-GTTCAGTGTCATCCCCCAGG-3'	MTRR_3_R	5'-TGCAGTCACCTCCATCACTG-3'	6538
MTRR	MTRR_4_F	5'-AGAGCACTGCGTCCTTTTGA-3'	MTRR_4_R	5'-GAAGCAACCAACAAGGCCAG-3'	7045
MTRR	MTRR_5_F	5'-TTCTCCCTGTGGCCTTCATT-3'	MTRR_5_R	5'-CACTGTCCTCGCCAAACAAA-3'	6173
MTRR	MTRR_6_F	5'-ATCGTGCCTAAGTCGGGTAG-3'	MTRR_6_R	5'-AGAAAAGGTCCCTCACTGCT-3'	4703
TPMT	TPMT_1_F	5'-TTTCACACAAACTTGAACTGTC-3'	TPMT_1_R	5'-GAGGTATCCGTCCCTGGG-3'	1865
TPMT	TPMT_2_F	5'-ATCCACTCACATCACCCTACATTATAAGCC-3'	TPMT_2_R	5'-GCTTCTTATAGATGAAATCACACACAAGCA-3'	6113
TPMT	TPMT_3_F	5'-CCGGCCACAAGTATGGACTT-3'	TPMT_3_R	5'-ACGAGGAACCAATGTGGCTAA-3'	6093
TPMT	TPMT_4_F	5'-AGGCAGGGCAAGGCATTTAT-3'	TPMT_4_R	5'-ACACAGGCATCCAAGGTCAG-3'	6004
CYP3A5	CYP3A5_1_F	5'-GCCTTTGGGTCCAGTGAAGA-3'	CYP3A5_1_R	5'-CCGAGACGCACCATTACACT-3'	6233
CYP3A5	CYP3A5_2_F	5'-AGACAAGACTGCACGTCTTTTCAAATATAC-3'	CYP3A5_2_R	5'-AAGTGAGTTAAACCTGTGTCTTCAAGTTGA-3'	8247
CYP3A5	CYP3A5_3_F	5'-TATCTTTACTATGTTCAGCCTTTCATCCCG-3'	CYP3A5_3_R	5'-CAGTTTCTTTCGAATTCTGGGAGTCAATCA-3'	5514
CYP3A5	CYP3A5_4_F	5'-CATGAAGATCACCACAACTAATGTGAGAAA-3'	CYP3A5_4_R	5'-GGCCATCTTCTCGCCACACTCCTTTTTTTT-3'	6925
CYP3A5	CYP3A5_5_F	5'-AGCCCTGGCATCAGCTATTT-3'	CYP3A5_5_R	5'-TGTGAGGCAGACACAAAGGG-3'	7066
CYP3A4	CYP3A4_1_F	5'-AACCTGTGGTTTTTAGAAGCTCCCCATGCA-3'	CYP3A4_1_R	5'-GCAGGATCCCACCAGTGAGAGGATTCATCC-3'	7418
CYP3A4	CYP3A4_2_F	5'-GGATGCAAGGCTGGGTTCAA-3'	CYP3A4_2_R	5'-AACAGGAGGGAATGTGGACA-3'	7270
CYP3A4	CYP3A4_3_F	5'-ACACACGCTACACTTCAGCA-3'	CYP3A4_3_R	5'-ACGCTGACTTGATCCTGGTG-3'	7838
CYP3A4	CYP3A4_4_F	5'-GCAGTAAAAACAGGTGTAATCTGAGTTTGT-3'	CYP3A4_4_R	5'-ACACCCAGGGCCAGGCTCCGTCAGACTACA-3'	6070
CYP3A4	CYP3A4_5_F	5'-GATTTGAGGGCTTCACTTAGATTTCTCTTC-3'	CYP3A4_5_R	5'-CAAGTCATTTGGAAATGTCATTTGGAAGTC-3'	8033
NAT2	NAT2_1_F	5'-CACCCGTACCAAGGTTCCTC-3'	NAT2_1_R	5'-TCTGGCTACAAGTCTGCAAAGT-3'	7073
NAT2	NAT2_2_F	5'-AGCTGTTGTGTTTGAAGTGGT-3'	NAT2_2_R	5'-GGCCAGATCTCCTATCCACC-3'	3173
